# Supplementary material for: Hot spot prediction in protein-protein interactions by an ensemble system
Source: BMC Syst Biol. 2018 Dec 31;12(Suppl 9):132. doi: 10.1186/s12918-018-0665-8 (PMC6311905; doi:10.1186/s12918-018-0665-8)
Supplement: Supplementary file 2 — All relevant MATLAB code. A simple Matlab implement of our predictor. (ZIP 19,742 kb) [file 12918_2018_665_MOESM2_ESM.zip › Additional file 2/Read ASA/ASA Property/BID/1JATB.txt]

 NetSurfP - Protein Surface Accessibility and Secondary Structure Predictions

  

|  |  |  |
| --- | --- | --- |
|  |  | NetSurfP - Protein Surface Accessibility and Secondary Structure Predictions  Technical University of Denmark |

  


---

```
# For publication of results, please cite:
# A generic method for assignment of reliability scores applied to solvent accessibility predictions.
# Bent Petersen, Thomas Nordahl Petersen, Pernille Andersen, Morten Nielsen and Claus Lundegaard
# BMC Structural Biology 2009, 9:51 doi:10.1186/1472-6807-9-51
#
# Column 1: Class assignment - B for buried or E for Exposed - Threshold: 25% exposure, but not based on RSA
# Column 2: Amino acid
# Column 3: Sequence name
# Column 4: Amino acid number
# Column 5: Relative Surface Accessibility - RSA
# Column 6: Absolute Surface Accessibility
# Column 7: Z-fit score for RSA prediction
# Column 8: Probability for Alpha-Helix
# Column 9: Probability for Beta-strand
# Column 10: Probability for Coil
E H  1JATB                  1    0.831 151.086   0.375   0.003   0.003   0.994
B M  1JATB                  2    0.332  66.433  -2.235   0.109   0.005   0.886
E S  1JATB                  3    0.429  50.220  -1.244   0.109   0.005   0.886
E K  1JATB                  4    0.589 121.198  -0.221   0.502   0.002   0.495
E V  1JATB                  5    0.487  74.867  -0.464   0.600   0.003   0.397
E P  1JATB                  6    0.454  64.423  -0.342   0.858   0.002   0.139
B R  1JATB                  7    0.161  36.777  -0.901   0.923   0.002   0.076
E N  1JATB                  8    0.319  46.731  -0.167   0.970   0.001   0.030
E F  1JATB                  9    0.437  87.706   0.800   0.970   0.001   0.030
B R  1JATB                 10    0.246  56.357   1.366   0.970   0.001   0.030
B L  1JATB                 11    0.029   5.365   0.668   0.970   0.001   0.030
B L  1JATB                 12    0.217  39.788   1.191   0.970   0.001   0.030
E E  1JATB                 13    0.459  80.170   1.608   0.970   0.001   0.030
B E  1JATB                 14    0.093  16.212   0.263   0.970   0.001   0.030
B L  1JATB                 15    0.082  15.069   0.828   0.988   0.000   0.012
E E  1JATB                 16    0.447  78.178   1.546   0.988   0.000   0.012
E K  1JATB                 17    0.476  97.831   2.216   0.970   0.001   0.030
B G  1JATB                 18    0.039   3.069  -0.033   0.923   0.002   0.076
E E  1JATB                 19    0.333  58.245   0.218   0.858   0.002   0.139
E K  1JATB                 20    0.702 144.422   0.452   0.694   0.003   0.303
E G  1JATB                 21    0.469  36.942  -1.258   0.246   0.004   0.750
E F  1JATB                 22    0.431  86.582  -0.767   0.016   0.005   0.979
E G  1JATB                 23    0.324  25.499  -1.294   0.003   0.003   0.994
E P  1JATB                 24    0.535  75.902  -2.256   0.005   0.015   0.979
E E  1JATB                 25    0.583 101.780  -1.979   0.005   0.045   0.951
B S  1JATB                 26    0.115  13.443  -2.060   0.005   0.262   0.733
B C  1JATB                 27    0.131  18.463  -0.624   0.004   0.616   0.381
B S  1JATB                 28    0.149  17.521  -1.204   0.002   0.816   0.182
B Y  1JATB                 29    0.125  26.819  -0.314   0.002   0.816   0.182
B G  1JATB                 30    0.142  11.152  -0.355   0.002   0.816   0.182
B L  1JATB                 31    0.207  37.810  -0.382   0.003   0.718   0.279
B A  1JATB                 32    0.268  29.545  -1.591   0.004   0.420   0.576
E D  1JATB                 33    0.372  53.533  -0.660   0.005   0.262   0.733
E S  1JATB                 34    0.452  53.033  -1.409   0.019   0.141   0.840
E D  1JATB                 35    0.509  73.405  -1.810   0.018   0.019   0.964
E D  1JATB                 36    0.476  68.577  -1.482   0.018   0.019   0.964
E I  1JATB                 37    0.360  66.619  -1.465   0.018   0.047   0.935
B T  1JATB                 38    0.228  31.651  -0.057   0.004   0.197   0.799
B M  1JATB                 39    0.050   9.905   0.217   0.004   0.514   0.481
B T  1JATB                 40    0.105  14.605   0.307   0.002   0.816   0.182
B K  1JATB                 41    0.194  39.906   1.197   0.001   0.900   0.099
B W  1JATB                 42    0.022   5.387   1.263   0.001   0.959   0.040
B N  1JATB                 43    0.152  22.311   1.275   0.000   0.983   0.017
B G  1JATB                 44    0.013   1.062   1.320   0.000   0.983   0.017
B T  1JATB                 45    0.079  11.013   0.431   0.000   0.983   0.017
B I  1JATB                 46    0.020   3.645   1.068   0.000   0.983   0.017
E L  1JATB                 47    0.214  39.220   0.786   0.001   0.900   0.099
B G  1JATB                 48    0.047   3.707  -0.577   0.004   0.514   0.481
E P  1JATB                 49    0.296  42.059  -0.333   0.004   0.138   0.858
E P  1JATB                 50    0.520  73.859  -1.367   0.005   0.045   0.951
E H  1JATB                 51    0.493  89.604  -0.813   0.005   0.045   0.951
B S  1JATB                 52    0.108  12.658  -1.323   0.005   0.015   0.979
B N  1JATB                 53    0.162  23.673  -0.752   0.018   0.088   0.893
B H  1JATB                 54    0.113  20.609  -0.535   0.018   0.088   0.893
E E  1JATB                 55    0.434  75.750  -0.734   0.018   0.088   0.893
E N  1JATB                 56    0.553  81.003  -1.801   0.004   0.085   0.910
B R  1JATB                 57    0.114  26.014  -1.035   0.005   0.336   0.660
B I  1JATB                 58    0.237  43.882   0.356   0.001   0.900   0.099
B Y  1JATB                 59    0.054  11.625   0.803   0.001   0.959   0.040
B S  1JATB                 60    0.218  25.514   1.308   0.001   0.959   0.040
B L  1JATB                 61    0.022   4.028   1.023   0.001   0.959   0.040
B S  1JATB                 62    0.205  24.049   0.950   0.000   0.983   0.017
B I  1JATB                 63    0.033   6.160  -0.091   0.001   0.959   0.040
E D  1JATB                 64    0.297  42.841   0.765   0.001   0.900   0.099
B C  1JATB                 65    0.048   6.767   0.785   0.004   0.616   0.381
B G  1JATB                 66    0.166  13.056   0.144   0.004   0.085   0.910
E P  1JATB                 67    0.622  88.276  -0.888   0.005   0.015   0.979
E N  1JATB                 68    0.609  89.099  -0.503   0.018   0.019   0.964
B Y  1JATB                 69    0.092  19.660   1.169   0.018   0.019   0.964
B P  1JATB                 70    0.205  29.160  -1.160   0.018   0.019   0.964
B D  1JATB                 71    0.251  36.111  -1.274   0.018   0.088   0.893
E S  1JATB                 72    0.308  36.074  -0.901   0.020   0.205   0.775
B P  1JATB                 73    0.122  17.340   0.490   0.004   0.197   0.799
B P  1JATB                 74    0.034   4.782  -1.838   0.004   0.420   0.576
B K  1JATB                 75    0.120  24.643  -0.836   0.001   0.900   0.099
B V  1JATB                 76    0.016   2.444   1.052   0.001   0.959   0.040
B T  1JATB                 77    0.095  13.149  -0.137   0.001   0.959   0.040
B F  1JATB                 78    0.023   4.516   0.159   0.001   0.900   0.099
B I  1JATB                 79    0.084  15.595  -0.710   0.004   0.616   0.381
B S  1JATB                 80    0.047   5.473  -0.946   0.021   0.279   0.699
B K  1JATB                 81    0.158  32.542  -0.889   0.019   0.141   0.840
B I  1JATB                 82    0.071  13.117  -1.808   0.004   0.197   0.799
B N  1JATB                 83    0.099  14.552  -2.484   0.021   0.279   0.699
B L  1JATB                 84    0.057  10.455  -0.542   0.018   0.088   0.893
B P  1JATB                 85    0.047   6.698  -3.348   0.018   0.047   0.935
E C  1JATB                 86    0.278  39.003  -1.377   0.019   0.141   0.840
B V  1JATB                 87    0.222  34.060  -1.556   0.021   0.451   0.528
B N  1JATB                 88    0.261  38.152  -0.533   0.022   0.359   0.619
E P  1JATB                 89    0.443  62.862  -1.535   0.020   0.205   0.775
E T  1JATB                 90    0.590  81.791  -1.815   0.004   0.138   0.858
E T  1JATB                 91    0.571  79.212  -1.060   0.005   0.045   0.951
B G  1JATB                 92    0.191  15.040  -1.385   0.004   0.085   0.910
E E  1JATB                 93    0.346  60.394   0.229   0.004   0.616   0.381
B V  1JATB                 94    0.046   7.101   0.547   0.002   0.816   0.182
B Q  1JATB                 95    0.048   8.501   0.215   0.021   0.756   0.223
B T  1JATB                 96    0.065   9.057  -0.173   0.021   0.451   0.528
E D  1JATB                 97    0.280  40.377  -0.839   0.021   0.279   0.699
B F  1JATB                 98    0.093  18.665  -0.539   0.018   0.088   0.893
B H  1JATB                 99    0.269  49.004  -0.637   0.018   0.047   0.935
E T  1JATB                100    0.458  63.497   0.120   0.018   0.019   0.964
E L  1JATB                101    0.487  89.151  -1.075   0.018   0.019   0.964
E R  1JATB                102    0.693 158.720  -0.552   0.018   0.019   0.964
E D  1JATB                103    0.642  92.541   0.429   0.005   0.015   0.979
B W  1JATB                104    0.048  11.664  -0.162   0.018   0.019   0.964
E K  1JATB                105    0.312  64.137   1.021   0.018   0.019   0.964
B R  1JATB                106    0.228  52.143  -0.464   0.257   0.016   0.727
E A  1JATB                107    0.361  39.793  -0.787   0.430   0.016   0.555
B Y  1JATB                108    0.209  44.663   0.889   0.268   0.043   0.689
B T  1JATB                109    0.216  29.973   0.626   0.257   0.016   0.727
B M  1JATB                110    0.021   4.142   0.909   0.938   0.007   0.055
B E  1JATB                111    0.244  42.679   1.000   0.975   0.003   0.022
E T  1JATB                112    0.324  44.883   1.405   0.975   0.003   0.022
B L  1JATB                113    0.025   4.504   1.326   0.975   0.003   0.022
B L  1JATB                114    0.023   4.193   1.260   0.970   0.001   0.030
B L  1JATB                115    0.225  41.124   1.197   0.970   0.001   0.030
B D  1JATB                116    0.105  15.145   0.199   0.970   0.001   0.030
B L  1JATB                117    0.016   3.003   1.613   0.988   0.000   0.012
B R  1JATB                118    0.149  34.052   0.840   0.988   0.000   0.012
E K  1JATB                119    0.289  59.365  -0.239   0.970   0.001   0.030
B E  1JATB                120    0.048   8.421  -0.134   0.923   0.002   0.076
B M  1JATB                121    0.026   5.183   0.281   0.782   0.003   0.216
E A  1JATB                122    0.317  34.933  -0.264   0.406   0.004   0.590
E T  1JATB                123    0.351  48.642   0.036   0.109   0.005   0.886
B P  1JATB                124    0.329  46.728  -0.845   0.109   0.005   0.886
E A  1JATB                125    0.383  42.240  -0.082   0.016   0.005   0.979
B N  1JATB                126    0.229  33.496  -1.287   0.016   0.005   0.979
E K  1JATB                127    0.670 137.798  -0.576   0.016   0.005   0.979
E K  1JATB                128    0.395  81.272   0.307   0.016   0.005   0.979
B L  1JATB                129    0.100  18.383  -0.841   0.053   0.005   0.942
B R  1JATB                130    0.215  49.189  -1.126   0.016   0.005   0.979
E Q  1JATB                131    0.380  67.814   0.314   0.053   0.005   0.942
E P  1JATB                132    0.337  47.863   0.660   0.321   0.003   0.675
E K  1JATB                133    0.674 138.724  -0.414   0.406   0.004   0.590
B E  1JATB                134    0.162  28.354  -1.265   0.502   0.002   0.495
B G  1JATB                135    0.205  16.110  -1.390   0.522   0.016   0.462
E E  1JATB                136    0.680 118.813   1.028   0.522   0.016   0.462
E T  1JATB                137    0.402  55.799   1.146   0.430   0.016   0.555
E F  1JATB                138    0.330  66.211  -0.722   0.016   0.005   0.979

Explain the output.  Go back.
```

---
